# To What Extent Do Childhood Interpersonal Relationships Influence Interest in a Potential Partner? An Experimental Study on a Sample of Young Adults

**DOI:** 10.1002/ijop.70103

**Published:** 2025-08-28

**Authors:** Martina Medolla, Anna Ferrara, Vincenzo Paolo Senese

**Affiliations:** ^1^ Department of Psychology University of Campania “Luigi Vanvitelli” Caserta Italy; ^2^ Universidad Nacional de Educacion a Distancia (UNED) Madrid Spain

**Keywords:** attachment theory, childhood relationships, interpersonal acceptance‐rejection theory, partner choice, sex differences

## Abstract

The objective of the present study was to examine the initial stages of partner selection in adulthood and assess the link between childhood interpersonal experiences with parents and the inclination toward intimacy with a potential partner. To achieve this goal, we devised an experimental paradigm to explore the initial mechanisms that guide interest toward one potential partner over another. Subsequently, we conducted a study involving a cohort of young adults (*N* = 200; aged 18–31) who were single, aiming to investigate their inclination toward intimacy with partners characterised by different internal working models (IWMs) and to assess the correlation with the quality of parent–child relationships during childhood. Additionally, aligning with existing literature and recognising the importance of exploring the moderating effect of sex on these processes, our study included both male and female participants matched by age. The results revealed, for the first time, that the initial propensity for intimacy with a partner is influenced by the interaction of multiple factors, including the partner's internal working model, the individual's internal working model, and their biological sex. The theoretical implications of these findings were discussed.

Numerous psychological studies have shown that humans possess an inherent tendency, desire, or need to form interpersonal relationships and to belong to social communities, a predisposition evident in germinal form in the neonatal stage and persisting throughout the lifespan (Baumeister and Leary 1995; Bowlby 1969, 1973, 1988; Rohner 2021). Erikson (1963), in his theory of psychosocial development, highlighted that during early adulthood, corresponding to the sixth developmental stage, individuals face the psychosocial crisis of establishing significant and meaningful intimate relationships versus experiencing isolation. In this phase, young adults are challenged to form intimate bonds; successful resolution results in strong relational ties, whereas failure may lead to loneliness and social isolation (Senese et al. 2021).

From this perspective, the capacity to establish stable and enduring interpersonal relationships—specifically romantic partnerships—is not only a fundamental social process (Holmes and Johnson 2009) but also a critical factor positively correlated with psychological well‐being and mental health (Feeney and Collins 2015). Consequently, elucidating the mechanisms underlying the formation of romantic relationships with a partner represents a significant objective in psychological research.

According to Attachment Theory (Bowlby 1973), partner choice is, among other factors, related to the quality of early interactions with primary caregivers. Indeed, the repetition of multiple interactive exchanges with primary attachment figures leads to the development of mental representations or Internal Working Models (IWMs) that guide expectations about the self and others in social relationships; these models developed in the early years are maintained throughout life (Bowlby 1973; Hazan and Shaver 1987). The American Psychological Association (2024) Dictionary defines the different profiles of individuals with different attachment styles: secure, a person who sees himself as worthy of love and others as reactive; dismissive, a person who has a model of himself as worthy of love and a model of the other as untrustworthy; fearful, a person who has little confidence in his/her own and others' skills; if they are in difficulty, they do not seek help; preoccupied, a person who has a negative internal model of himself and a positive one of others. A seminal study investigated the relationship between attachment styles and intimate relationships in adulthood, showing that adults with discomfort with closeness have greater difficulty in establishing stable and lasting romantic relationships (Sagone et al. 2023).

Similarly, according to the Interpersonal Acceptance‐Rejection Theory (IPARTheory; Rohner 2021) the quality of primary childhood relationships plays a crucial role in shaping adults' psychological adjustment and interpersonal relationships. Indeed, in line with the theory, any interpersonal relationships can be placed somewhere on a *continuum*, the interpersonal acceptance and rejection dimension, that reflects the extent to which the individual feels accepted (or rejected) in the relationship. In this view, the psychological adjustment in children and adults and the quality of adult romantic relationships would be related to the degree of perceived acceptance in important relationships. Recently, this hypothesis has been supported by a multicentre cross‐cultural study, involving 13 different countries, showing a significant association between perceived acceptance by mother and father and fear of intimacy in adult relationships, and that this effect was only partially mediated by the general psychological well‐being (Rohner et al. 2019).

In summary, although previous research has established the impact of childhood interpersonal experiences on personality development and satisfaction in intimate relationships, the extent to which these experiences influence the initial stages of partner selection remains unclear. This gap likely reflects the absence of a suitable paradigm to study early partner choice. To address this, we first developed an experimental procedure to examine the mechanisms guiding initial interest in potential partners; then applied it in a study involving young single adults (aged 18–31) to assess propensity for intimacy toward partners characterised by different IWMs and evaluate the association with the quality of childhood interpersonal experiences.

The main objectives of the study were to (1) develop an experimental procedure to study the first steps in choosing a partner; and (2) evaluate the association between childhood experiences and propensity to intimacy toward a possible partner. Moreover, since there are studies (Monteoliva et al. 2012) that have highlighted sex differences in the desired traits of a partner and in the relationship dynamics, we analysed data separately for male and female participants and tested the moderation effect of sex to investigate to what extent the processes that influence the intention to choose a partner are similar across sexes.

To address the first objective—examining factors influencing the early‐stage selection of a potential partner—participants were presented with four descriptive profiles, each representing a prototypical attachment style based on established literature. The profiles were gender‐neutral to avoid bias related to sexual preference, and participants were asked to imagine each profile as describing someone they found physically attractive. Following each profile, participants rated the type and depth of relationship they would desire with the individual, using a set of ad hoc items assessing their propensity for intimacy. This construct was defined as the level of intimacy participants would ideally seek or consider based on the profile description. The procedure was designed to reflect contemporary relational dynamics, such as those found in online dating contexts, where individuals make relational decisions based on limited information.

To address the second objective—validly assessing the influence of childhood interpersonal experiences on partner preference—we adopted a dual‐theoretical approach, drawing on both Attachment Theory (Feeney et al. 1994) and Interpersonal Acceptance‐Rejection Theory (IPARTheory; Rohner 2005). In line with Attachment Theory, we measured IWMs of participants to explore potential continuity between individuals' cognitive‐affective representations of the self and others developed through early attachment experiences and the characteristics of preferred partners. From the perspective of IPARTheory, we assessed recollections of parental rejection from both mother and father to examine the extent to which the perceived quality of these early and specific relationships was associated with partner preferences. This dual approach also enabled us to pursue two additional aims: to investigate the associations between IWMs and parental acceptance‐rejection experiences and to examine the distinct effects of maternal and paternal relationship quality on individuals' propensity for intimacy with a potential partner.

Considering the previous literature and our objectives, we expected: (1) a significant difference in propensity to intimacy toward possible partners in function of the type of partner profile, for example, secure profile should be chosen more than other profiles; (2) a significant association between IWMs and interest toward possible partners, for example, those who are confident in relationships, which reflects a secure type of attachment (Feeney et al. 1994), should tend more to consider a partner with a secure profile than other partners with a different IWMs; (3) a significant association between perceived acceptance‐rejection with each of the two parents and propensity to intimacy toward possible partners, for example, those who reported a lower rejection from parents should tend more to choose a partner with a secure profile than other profiles, respect for those who reported a higher rejection from parents; and (4) a significant association between IWMs and acceptance‐rejection experiences with each of the two parents. Finally, sex differences in the relationship dynamics were expected, although we did not have a specific expectation of the type of moderation expected.

## 1 Method

### 1.1 Participants

The sample consisted of 200 single participants, 111 women and 89 men. The age range of participants was from 18 to 31 years (*M* = 24.0 years; SD = 2.80). Males (*M* = 24.0 years; SD = 3.04) and females (*M* = 24.0 years; SD = 2.66) participants were matched in terms of age (*F* < 1). Participants were eligible for the study if they were not currently in a stable relationship and were aged between 18 and 31 years. The protocol was created and administered online through Google Forms. A convenience sampling procedure was used. Recruitment took place both through in‐person recruitment and using social networks. Participants with the inclusion criteria were introduced to the purpose of the research and were asked if they wanted to join the research project. To those available to participate, the link to the protocol was sent by e‐mail with instructions on how to fill in the protocol. The timeline for data collection was from February 2022 to August 2022. Data was collected in accordance with Articles 13 and 14 of the EU General Data Protection Regulation No. 679/2016 and the Declaration of Helsinki. All participants signed an online informed consent before starting data collection. The data were collected completely anonymously and in line with the requirements of the ethics committee of the authors' department.

### 1.2 Procedure

The study followed a within‐subjects experimental design, in which each participant was exposed to all four partner profiles (Secure, Dismissing, Fearful/Preoccupated, and Disorganised or Unresolved), presented in random order to avoid order and sequence effects. Regarding the analysis of the associations between parental relational experiences, the IWMs, and partner choice, the relevant research design is correlational in nature. In detail, the protocol included the following measures: a sociodemographic questionnaire, to collect basic personal information; two short forms of the adult Parental Acceptance‐Rejection Questionnaire, to evaluate remembrances of the perceived acceptance‐rejection toward the mother and the father respectively (Rohner 2021); the Attachment Style Questionnaire, to evaluate participants' IWMs (Feeney et al. 1994); and the preference of partner procedure to evaluate the propensity to intimacy toward each potential partner. The time to complete the protocol was approximately 30 min for each participant.

### 1.3 Measures

#### 1.3.1 Propensity to Intimacy

To assess the propensity to intimacy toward a partner, we created four profiles (see Table 1), describing individuals in a neutral manner, that is, valid for both men and women, characterised by a specific attachment style (see Main and Goldwyn 1984, 1998; Hesse 1999): (1) Secure or Free [F], (2) Dismissing [Ds], (3) Fearful/Preoccupated [E], and (4) Disorganised or Unresolved [U]. The validity of the profiles was verified by presenting them to two independent and blind experts, who classified each profile according to the attachment categories. The results confirmed the congruence between the categories assigned by the experts and the reference categories. Once the profiles were defined, five items were developed and associated with each profile, to measure the propensity to intimacy with that potential partner. In particular, the following questions were considered: “Would you go out for tea/coffee with him/her?”; “Would you enter a relationship of sexual interest with him/her?”; “Would you have a romantic relationship with him/her?”; “Would you introduce him/her to your family?”; “Would you start a family with him/her?”. Thus, each participant was presented with the individual profiles in random order and for each one, the five statements were presented. For each item participants were asked to respond using the 3‐point scale: “No, not at all” (=1); “Probably yes” (=2); “Definitely yes” (=3). To verify the measurement model of the five items of propensity to intimacy, a principal component analysis (PCA) was first performed on the responses observed for each profile. The results confirmed the presence of a one‐dimensional structure in the responses. In all cases, results showed that the eigenvalue of the first component is greater than 3.0 and accounts for more than 61% of the variance. Therefore, a percentage index of the propensity for intimacy was computed for each profile: the observed responses to the five items were first summed, then the result was divided by the total theoretical score of the scale (i.e., 15), and the result was multiplied by 100. The reliability analysis, assessed by means of Cronbach's alpha index, showed adequate reliability for all calculated indices (*α*s > 0.86).

**TABLE 1:** Profiles of the four different potential partners.

Attachment style	Description
Secure or free [F]	O is a friendly person who enjoys reading books, especially those related to art, and loves going to the beach and taking long walks. Early morning runs are part of their routine, which they consider beneficial for their health. They enjoy spending time with friends and going on trips to explore the world. In relationships, they tend to consider themselves worthy of love and perceive others as sensitive and open toward them. They are not afraid of intimacy and consistently pursue their own thoughts and ideas. Emotional closeness and attachment to significant others are approached without fear of abandonment. Their relationships are generally stable and based on trust. They love overcoming their fears and put themselves on the line to confront and surpass them. They are usually able to connect easily with others and sees themselves as a pleasant and respectable individual.
Dismissing [Ds]	O is a very driven person. Working is considered essential in life for them to feel independent. In their free time, tension is relieved by going to the gym. They have a positive self‐image. They value independence and self‐sufficiency, so they handle many problematic situations alone, without asking for help, even when support might be needed. They would like others to do the same, as depending on others or having others depend on them is not appreciated. At the end of the day, relaxation sometimes comes through baking a cake and listening to calming music.
Fearful/Preoccupated [E]	O is a person who enjoys being surrounded by people, and a vital part of their life is their friends. They try to please others and avoid disappointing them, and other people's opinions are important to them. They love hosting dinners. When it comes to making decisions, they tend to act condescendingly. Behaviourally, they differ in hypersensitivity and extreme need for attention. They don't believe in themselves but trust other people's abilities. Their passion for wine has led them to visit many wine cellars and get to know many people from various parts of the world, and this has allowed them to travel a lot.
Disorganised or Unresolved [U]	O is a person who loves nature to the point of wanting to be one with it. When they are lost in their thoughts, they appreciate long walks in green areas. In relationships, they desire intimacy and social contact, but they don't trust others and fear rejection, so they avoid social situations. The idea of being unloved is deeply rooted in them. Their passion is photography and go on excursions in pursuit of this hobby.

*Note*: The letter “O” neutrally denotes the reference partner; the pronoun “they” is used as a singular, gender‐neutral form to refer to individuals.

#### 1.3.2 Parental Acceptance‐Rejection

To assess adults' remembrances of parental acceptance‐rejection experienced in childhood, the Italian short version of the adult Parental Acceptance‐Rejection Questionnaire (Rohner 2005; Senese et al. 2016) was used. The 24‐item version of the self‐report scale yields scores on four scales: (1) warmth/affection; (2) hostility/aggression; (3) indifference/neglect; and (4) undifferentiated rejection. Items are scored on a 4‐point scale from (4) almost always true through (1) almost never true. The sum of the four scales (with the warmth/affection scale reverse scored to create a measure of coldness/lack of affection) constitutes a measure of overall perceived maternal and paternal rejection. Extensive evidence in Khaleque and Rohner (2002) and Rohner (2005) shows the adult Parental Acceptance‐Rejection Questionnaire to be a reliable and valid measure (see also Senese et al. 2016). The reliability analysis, assessed by means of Cronbach's alpha index, showed adequate reliability for all calculated indices and the total score (*α*s > 0.83).

#### 1.3.3 Internal Working Model

To assess individual differences in adult attachment, the Italian version of the Attachment styles Questionnaire was used (Feeney et al. 1994). The Attachment styles Questionnaire is a 40‐item self‐report scale that yields scores on five scales or dimensions: (1) Trust; (2) Discomfort for Intimacy; (3) Secondary Relationships; (4) Need Approval; and (5) Worry for Relationships. Each item describes an attachment prototype description. For each item, participants are asked to indicate the degree to which the propositions describe their feelings on a 6‐point scale, from totally disagree (1) to totally agree (6). The reliability analysis, assessed by means of Cronbach's alpha index, showed adequate reliability for all calculated indices (*α*s > 0.80).

### 1.4 Data Analysis

Preliminary descriptive analyses were executed to investigate missing values and variables' distributions. Univariate distributions of observed variables were examined for normality. These analyses indicated that there were no missing values or normality problems. In line with the objectives, to investigate if the propensity to intimacy was affected by the attachment profile of the partner and the sex of the participant, a 4 × 2 mixed ANOVA was carried out on the propensity to intimacy score, with the kind of attachment profile as a 4‐level within‐subject factor and the participant sex as a 2‐level between‐subject factor. The Bonferroni correction was used for post hoc comparisons to control the familywise Type I error; whereas the effect size of significant effects was expressed by partial eta squared (*η* ^2^_p_).

To investigate if there is a continuity between childhood interpersonal experiences and the interest toward a possible partner and the moderation effect of participants' sex on this association, correlation coefficients were computed separately for male and female participants. In particular, the association between the participants' IWMs and the propensity to intimacy toward a possible partner and between the remembrances of maternal and paternal acceptance or rejection and the propensity to intimacy toward a possible partner were computed as a function of each profile. Finally, to verify the degree of association between IWMs and acceptance‐rejection experiences with each of the two parents, the association between Attachment styles Questionnaire scores and the adult Parental Acceptance‐Rejection Questionnaire total score was also computed. To test the potential moderating effect of sex on the computed coefficients, a moderation analysis was conducted using the PROCESS macro v. 4.2 (Hayes 2022), estimating the significance of the effect through the bootstrap method. Specifically, to minimise the risk of Type I error, this analysis was performed exclusively on the correlation coefficients that were found to be statistically significant.

## 2 Results

**FIGURE 1:**
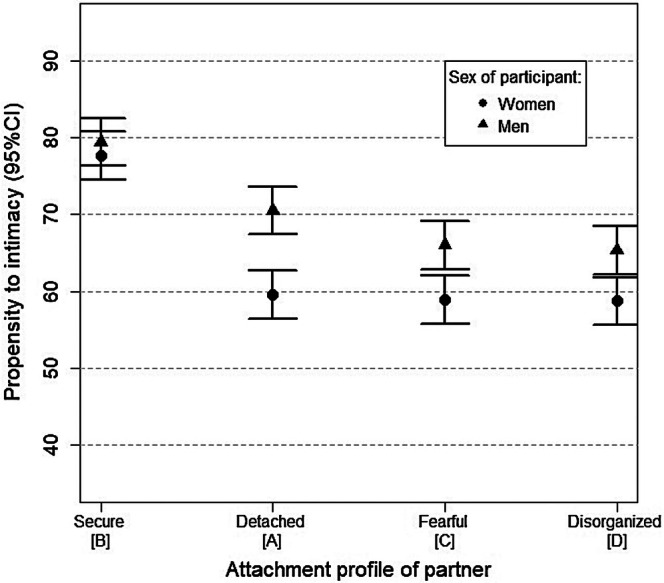
Mean propensity to intimacy with potential partners as a function of sex of participant and attachment profile.

The 4 × 2 mixed ANOVA on the propensity to intimacy score showed that the interest toward a possible partner was influenced by the profile of the partner (*F*(3, 594) = 56.25, *p* < 0.001, η^2^_p_ = 0.22), participant sex (*F*(1, 198) = 17.88, *p* < 0.001, η^2^_p_ = 0.08), and their interaction (*F*(3, 594) = 3.32, *p* = 0.02, η^2^_p_ = 0.02). Post hoc analysis indicated that, independently of the sex of the participant, the “Secure or Free” profile (*M* = 78.50, *p* < 0.001) showed higher score in propensity to intimacy than “Detached/Devaluating or Dismissing” (*M* = 65.0), “Fearful” (*M* = 62.5) and “Concerned/Disorganized or Unresolved” (*M* = 62.0). The effect size was large. Men showed a greater propensity to intimacy (*M* = 70.30) than women (*M* = 63.70) across all profiles. The effect size was medium. For the interaction, analysis showed no difference across men and women for the secure profile, whereas men had a significantly greater propensity for intimacy than women toward the three insecure profiles (see Figure 1). The effect size was small.

Correlation analysis revealed that in both women and men, those who had more confidence in relationship were less likely to prefer the partner with the “Fearful” profile, whereas the confidence in relationship was not associated with propensity to intimacy toward the other profiles (see Table 2). No other significant associations emerged, suggesting that other—not considered factors—could influence intimacy propensity in the two groups. In women, the propensity to intimacy toward the “Secure or Free” profile was negatively related to Secondary relationships score. For the “Fearful” profile, it was positively related to Intimacy discomfort, Need for approval, and Concern about relationships; and for the “Concerned/Disorganized or Unresolved” profile, it was positively related to Need for approval, and Concern about relationships. This seems to suggest that women tend to show continuity between insecure IWMs and preferences for partners with an insecure profile. In men, the propensity to intimacy toward the “Secure or Free” profile and the “Detached/Devaluating or Dismissing” partner was negatively related to Intimacy discomfort. This indicated less continuity in men between IWMs and partner characteristics, as shown by a general lower preference for partners with higher Intimacy discomfort, regardless of partner profile. This pattern was partially confirmed by moderation analysis, which found that sex moderated the association between Intimacy discomfort and the propensity to intimacy with the partner with the “Concerned/Disorganised or Unresolved” profile (*p* = 0.013). In all other cases, the results approached significance but did not reach statistical significance at the specified *α* level.

**TABLE 2:** Correlation between attachment styles questionnaire subscales and propensity to intimacy with potential partners as a function of sex of participant and attachment profile.

	Attachment profile					
ASQ	Secure (B)	Detached (A)	Fearful (C)	Disorganised (D)	*M*	SD
Women						
1. Confidence in relationship	0.13	0.08	−0.18*	0.02	29.99	5.43
2. Intimacy discomfort	−0.11	0.05	0.23**	0.03	40.68	7.81
3. Secondary relationships	−0.21*	0.04	−0.01	0.05	15.19	5.86
4. Need of approval	0.03	0.01	0.28***	0.24**	24.85	7.42
5. Concern about relations	0.09	−0.09	0.19*	0.22*	31.33	7.13
M (SD)	77.60 (1.60)	59.60 (1.50)	58.92 (1.64)	58.74 (1.66)		
Men						
1. Confidence in relationship	0.11	0.06	−0.18*	0.10	30.57	7.00
2. Intimacy discomfort	−0.23*	−0.14	−0.01	−0.26**, ^a^	39.75	7.46
3. Secondary relationships	−0.10	0.18*	−0.10	−0.09	19.38	6.82
4. Need of approval	0.14	0.00	0.09	0.04	24.09	8.44
5. Concern about relations	0.16	−0.06	0.04	0.04	30.79	8.00
M (SD)	79.40 (1.77)	70.49 (1.64)	65.99 (1.83)	65.32 (1.85)		

Abbreviation: ASQ, attachment styles questionnaire.

^a^ The moderating effect of sex was statistically significant, *p* = 0.013.

* *p* < 0.05.

** *p* < 0.01.

*** *p* < 0.001.

Although the procedure developed to assess partner preferences is newly designed and has not been previously validated, these initial analyses support its construct validity. Participants consistently showed the highest propensity to intimacy toward the “Secure or Free” profile, as expected based on theoretical models of attachment and prior literature. Moreover, the associations between participants' IWMs and preferences for insecure profiles (e.g., “Fearful,” “Concerned/Disorganized or Unresolved”) reflected coherent and theoretically grounded differences between male and female participants. These findings suggest that responses to the profiles align with attachment theory predictions, providing preliminary evidence for the procedure's validity.

About the correlation between the remembrances of maternal and paternal acceptance or rejection and the propensity to intimacy toward a possible partner, no significant associations were observed (see Table 3). Similarly, no differences were observed in the associations between men and women.

**TABLE 3:** Correlation between PARQ scales and propensity to intimacy with potential partners as a function of sex of participant and attachment profile.

	Attachment profile					
Scale	Secure (B)	Detached (A)	Fearful (C)	Disorganised (D)	*M*	SD
Women						
1. PARQ Mother	−0.10	−0.02	−0.05	−0.00	46.73	11.67
2. PARQ Father	−0.04	0.10	−0.09	−0.15	51.02	13.49
Men						
1. PARQ Mother	−0.02	−0.03	−0.06	−0.04	49.28	12.42
2. PARQ Father	0.04	0.02	−0.11	−0.06	53.16	13.93

*Note*: PARQ, Parental Acceptance‐Rejection Questionnaire; none of the correlation coefficients reported in the table were statistically significant at the *α* = 0.05 level.

Finally, there were clear differences between men and women in how their IWMs were related to their remembrances of maternal and paternal acceptance or rejection (see Table 4). Indeed, among men, those who remembered being accepted (low rejection) by both their parents had IWMs characterised by higher security and/or lower insecurity. Whereas, among women, there was less congruence between the remembrances of maternal and paternal acceptance and the IWMs, with only a weak association between acceptance‐rejection experiences and IWMs. This latter finding seems to indicate the presence of other factors besides those considered responsible for IWMs in women. In this case, while the moderation analysis confirmed in several cases the statistical significance of the differences between correlations observed in men and women, there were also multiple cases in which the moderating effect approached but did not reach significance. Specifically, significant sex differences emerged for the association between the Secondary relationships score and recollections of parental acceptance, both maternal (*p* = 0.020) and paternal (*p* = 0.004), as well as for the association between the score on relationship anxiety and recollections of paternal acceptance (*p* = 0.035).

**TABLE 4:** Correlation between attachment styles questionnaire subscales and parental acceptance‐rejection questionnaire scales as a function of sex of participant.

	PARQ	
ASQ	Mother	Father
Women		
1. Confidence in relationship	−0.19*	−0.18*
2. Intimacy discomfort	0.15	0.17*
3. Secondary relationships	0.01	0.06
4. Need of approval	0.13	0.07
5. Concern about relations	0.16	0.09
Men		
1. Confidence in relationship	−0.43***	−0.38***
2. Intimacy discomfort	0.23*	0.36***
3. Secondary relationships	0.35***, ^a^	0.48***, ^b^
4. Need of approval	0.36***	0.36***
5. Concern about relations	0.35***	0.40***, ^a^

Abbreviations: ASQ, attachment styles questionnaire; PARQ, parental acceptance‐rejection questionnaire.

^a^ The moderating effect of sex was statistically significant, *p* < 0.05.

^b^ The moderating effect of sex was statistically significant, *p* < 0.1.

* *p* < 0.05.

*** *p* < 0.001.

## 3 Discussion

The objectives of the study were threefold: to develop an experimental procedure for investigating the initial stages of partner selection, to examine individuals' propensity for intimacy toward partners characterised by different IWMs, and to evaluate the association between such propensities and childhood interpersonal experiences. To achieve these aims, an experimental paradigm was implemented with a sample of young adults. The most noteworthy finding is that the procedure demonstrated preliminary validity, as participants' preferences for potential partners followed patterns consistent with those previously reported in the literature. Furthermore, the results indicated—for the first time—that the propensity for intimacy toward a potential partner is influenced by multiple factors, including the partner's characteristics, the individual's IWMs, and the biological sex of the person described. Notably, moderation analyses revealed statistically significant sex differences in a limited number of cases; whereas in several others, moderation effects approached—but did not meet—the conventional threshold for statistical significance.

Previous studies examining partner choice showed that partners with secure attachment styles were preferred over partners with other attachment styles (Latty‐Mann and Davis 1996). Our results confirm this finding; moreover, for the first time, they provide evidence that differences in partner preferences can be associated with sex and individual relational experiences. Sex differences in attachment styles may result from an interaction between the activation effects of adrenal androgens and the organisational effects of prenatal sex hormones (Del Giudice and Angeleri 2016). The literature (see for example Fletcher et al. 2004) has shown several results related to stable sex differences in partner desires and behaviour; for example, data indicate that men possess a stronger and less malleable sex drive than do women (Baumeister 2000; Baumeister et al. 2001); men are less adept and motivated in maintaining intimacy in close relationships than are women (Fletcher 2002), all data congruent with what was observed.

Additionally, we examined the extent to which childhood experiences and adult IWMs influence partner preference. Consistent with the literature (Holmes and Johnson 2009) on the relationship between adult IWMs and the propensity for intimacy with partners characterised by specific profiles, our findings confirm this association, albeit with some deviations from initial expectations. These differences were influenced not only by biological sex but also by the specific measure employed.

Regarding IWMs, significant associations emerged between preferences for different partner types; however, these associations varied between men and women. We interpret this as evidence of greater congruence—and thus continuity—in patterns of childhood interpersonal experiences, particularly among men compared to women. Moderation analyses supported these sex differences; although statistical significance was not achieved consistently across all comparisons.

Conversely, when considering recollections of specific relational qualities, the association with partner preference was no longer evident. This suggests that it is the IWMs, rather than specific childhood experiences per se, that mediate partner preference, and that this mediating process differs between men and women. In line with Fraley and Shaver's (2000) review, this finding can be understood within the framework that links childhood memories of parental acceptance or rejection to the formation of adult IWMs. Given the significant association between these dimensions, one might hypothesise, in agreement with prior research (Rohner et al. 2019), that the influence of parental acceptance experiences on intimacy propensity is mediated by IWMs, which act as proximal predictors alongside additional factors not explored in the present study.

## 4 Limitations and Conclusions

This study presents both strengths and limitations that should be acknowledged when interpreting the findings. Among the strengths is the adoption of a novel and structured approach to examining partner preference and its association with attachment‐related processes. The integration of experimental and correlational methodologies allowed for a multi‐dimensional analysis of a complex psychological construct. Additionally, the procedure developed to assess partner preference—although innovative and requiring further validation—yielded data consistent with theoretical expectations, offering preliminary support for its validity.

However, several limitations must be considered. From a theoretical perspective, a key limitation concerns the absence of relational context in interpreting partner preferences. According to Sexual Strategies Theory (Buss and Schmitt 2016), understanding partner selection requires consideration not only of biological sex and temporal context (e.g., short‐ vs. long‐term mating strategies) but also of ecological and individual factors such as partner value, sex ratio, and parasite prevalence. Future research should incorporate these dimensions to account for more nuanced patterns of preference.

Methodologically, the study's mixed design—combining experimental and correlational components—enabled both control and exploration; yet the correlational nature of some analyses limits causal interpretation. Longitudinal studies are recommended to examine the directionality of the observed associations. While the experimental component enhanced internal validity, it may have reduced ecological validity. Complementary methods, including observational or immersive virtual reality paradigms, may strengthen the real‐world applicability of future findings.

Regarding generalisability, the sample was recruited through ad hoc methods within a specific sociocultural context, which limits external validity. Broader and more diverse samples would enhance the robustness and replicability of the results. Although the associations found between childhood experiences, IWMs, and partner preferences align with established theories, the study did not investigate the underlying mechanisms. Future work should explore mediating and moderating variables to better understand these dynamics.

Finally, while the sample size provided adequate power for most statistical analyses, larger samples would allow for more sophisticated multivariate modelling and enhance the stability of results, particularly for findings approaching significance thresholds.

Overall, this study contributes valuable insights into the early mechanisms of partner selection, highlighting the interplay of individual attachment models, partner characteristics, and sex. Future cross‐cultural replications and extended designs will be crucial to deepen our understanding and explore additional influencing factors.

## Author Contributions

**Martina Medolla:** Conceptualization (equal); Formal analysis (supporting); Investigation (equal); Writing – original draft (equal); Writing – review & editing (equal); Project administration (equal). **Anna Ferrara:** Conceptualization (equal); Investigation (equal). **Vincenzo Paolo Senese:** Conceptualization (equal); Formal analysis (lead); Methodology (lead); Investigation (equal); Writing – review & editing (equal); Writing – original draft (equal); Project administration (equal); Supervision (lead).

## Ethics Statement

All procedures performed in studies involving human participants were in accordance with the ethical standards of the institutional research committee at University of Campania, Luigi Vanvitelli and with the 1964 Helsinki Declaration and its later amendments or comparable ethical standards.

## Consent

Informed consent was obtained from all individual adult participants included in the study.

## Conflicts of Interest

The authors declare no conflicts of interest.
